# Evolutionary adaptation of high‐diversity communities to changing environments

**DOI:** 10.1002/ece3.6695

**Published:** 2020-10-13

**Authors:** Evgeniia Alekseeva, Michael Doebeli, Iaroslav Ispolatov

**Affiliations:** ^1^ Skoltech Moscow Russia; ^2^ University of British Columbia Vancouver British Columbia Canada; ^3^ Universidad de Santiago de Chile (USACH) Santiago Chile

**Keywords:** adaptation, environmental changes, extinction

## Abstract

We use adaptive dynamics models to study how changes in the abiotic environment affect patterns of evolutionary dynamics and diversity in evolving communities of organisms with complex phenotypes. The models are based on the logistic competition model, and environmental changes are implemented as a temporal change of the carrying capacity as a function of phenotype. In general, we observe that environmental changes cause a reduction in the number of species, in total population size, and in phenotypic diversity. The rate of environmental change is crucial for determining whether a community survives or undergoes extinction. Until some critical rate of environmental changes, species are able to follow evolutionarily the shifting phenotypic optimum of the carrying capacity, and many communities adapt to the changing conditions and converge to new stationary states. When environmental changes stop, such communities gradually restore their initial phenotypic diversity.

## Graphical abstract

We use adaptive dynamics models to study how changes in the abiotic environment, such as climate change, affect patterns of evolutionary dynamics and diversity in evolving communities of organisms with complex phenotypes. We observe that the rate of environmental change is a crucial factor that determines whether the community survives and adapts to the changing conditions or undergoes extinction. In the majority of surviving systems, we observe a reduction in the number of species, total population size, and phenotypic diversity.

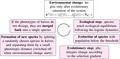

## 1 INTRODUCTION

Over the past decades, the issue of the impact of changing environmental conditions on species and ecosystems has gained increasing prominence, particularly in the context of global warming (Trisos, Merow, & Pigot, 2020). Recent estimates have shown that at the current rate of global warming, one of six species will become extinct (Urban, 2015), and empirical evidence supports this finding (Maclean & Wilson, 2011). Already there are species whose extinction occurred as a result of climate change. For example, the sea level rise has destroyed the habitat of mosaic‐tailed rat (*Melomys rubicola*) and individuals of this species have not been seen since 2009 (Gynther, Waller, & Leung, 2016). Many species, such as polar bears (*Ursus maritimus*), experience ecological stress. For hunting, this species relies on sea ice, where seals, their primary source of food, rest, and breed. Reduction in ice surfaces forces polar bears to overcome long distances by swimming and thus strongly affects the balance between anabolism and catabolism (Lone et al., 2018). Several studies detected muscle atrophy and weight loss in polar bears because of starvation and changes in metabolism of lipids (Griffen, 2018; Obbard et al., 2016; Pagano et al., 2018; Tartu et al., 2017; Whiteman et al., 2017).

However, the majority of ecosystems are characterized by extensive adaptability. While changing environmental factors often lead to diversity reduction, in general many ecosystems will likely survive. For example, this is currently observed in coral reefs. Increasing temperature and acidification of the ocean water affect the symbiotic relationships between corals and microalgae in such a way that corals expel their endosymbionts and bleach (Pogoreutz et al., 2018). Without benefits of symbiosis, corals experience higher mortality, become more sensitive to diseases, and decline. However, because strains of both host and endosymbiont vary in their sensitivity to higher temperatures, some of them can form a thermo‐tolerant symbiosis (Baker, 2003; Bay, Rose, Logan, & Palumbi, 2017; Grottoli et al., 2018; Little, Van Oppen, & Willis, 2004; Smith, Hume, Delaney, Wiedenmann, & Burt, 2017). Such examples already exist in zones with extreme temperatures, such as the Arabian/Persian Gulf (PAG) (Baker, Starger, McClanahan, & Glynn, 2004); hence, this symbiotic community can adapt, in principle, to changing environments despite a decrease in diversity of its participants. In a recent study of time series of species composition from various geographical areas, Blowes et al. (2019) made a prediction that with time climate change will mostly cause large‐scale reorganization of biodiversity rather than its global decline.

The challenges imposed by changing environments vary widely between ecosystems and between species within ecosystems, as, for example, the warming climate induces a diverse spectrum of interconnected changes in environmental conditions and weather patterns. Besides global climatic changes, there are numerous other examples of how anthropogenic activity disturbs ecosystems locally by environmental pollution, poaching, modification of geographical landscapes and many others factors (Laskar, Mahata, & Liang, 2016; Scheffers, Oliveira, Lamb, & Edwards, 2019). Adaptation to changing environments is also an important topic of research in the context of preventing the development of antibiotic and drug resistance. So, in one way or another, biological populations frequently face a changing environment, which is an important force in their evolution.

Adaptation to environmental changes has been the subject of both experimental and theoretical research. A nice example of an experimental study of evolution in an artificially created changing environment is the work on gradual bacterial adaptation to increasing doses of antibiotic on a giant Petri dish (Baym et al., 2016). Another example of experimental adaptation to a changing environment was observed in a study of phytoplankton biodiversity in increasingly warm water (Yvon‐Durocher et al., 2015). However, the slowness of evolutionary processes on human timescales sets strong restrictions of what can be done in such experiments.

Free of this limitation, theoretical studies are by far more numerous. For example in Botero, Weissing, Wright, and Rubenstein (2015), the authors found that certain types of climatic changes force populations to cross “tipping points” and to switch from one adaptive strategy to a qualitatively different one, which often leads to extinction despite the successful adaptation in the context of the previously used strategy. Another theoretical study has revealed the influence of genetic variance and spatial dispersal on the success of a given number of competing species subjected to changing conditions (Norberg, Urban, Vellend, Klausmeier, & Loeuille, 2012). The combination of high genetic variance and low spatial dispersal is the most conducive to adaptation and survival of the species under the effect of climatic change. In Jones (2008) and Northfield and Ives (2013), the authors have investigated how coevolution in pairs of species with various types of ecological interactions affects the process of adaptation to environmental changes. They argued that types of coevolution with conflicting interests help species to adapt, often counterintuitively, whereas types of coevolution with nonconflicting interests enhance the detrimental effect of climatic changes. These conclusions are corroborated by the studies specifically focused on predator–prey interactions (Mellard, de Mazancourt, & Loreau, 2015; Osmond, Otto, & Klausmeier, 2017), which demonstrate that under certain conditions, and especially when the predator–prey interaction trait is aligned with the direction of environmental change, predation increases adaptability and resilience.

The adaptive dynamics and individual‐based model described in Johansson (2008) and later adapted to different scenarios in many subsequent studies, predicts a decrease in diversity and possible complete extinction in the system of 1–3 competing species subject to an environmental change at a constant or fluctuating rate. An extension of the model (Johansson, 2008) was used to investigate multi‐faceted effects of asymmetric resource availability or competition between two species on the response to a changing environment (Van Den Elzen, Courtney, Kleynhans, & Otto, 2017). More complex scenarios of multi‐patch environment with migration (De Mazancourt, Johnson, & Barraclough, 2008) and cyclic changes in the environmental gradient (De Mazancourt et al., 2008) have also been investigated in the context of effects of the climate change on species diversity and population.

In this work, we take a somewhat different look at the influence of environmental changes on an evolving system. As is widely reported, the problem with environmental changes is often not so much the actual state of the environmental variable, such as the global temperature or the CO_2_ concentration, but the high and previously unseen rates at which these variables change. Thus, in this work we investigate how an ecosystem, modeled as a community of interacting and evolving species, reacts to environmental changes of various rates. Similarly to (Johansson, 2008), we focus on the particular case of competing species, ignoring for now other ecological interactions, and consider a diversifying community described by a logistic competition model. However, as an extension of (Johansson, 2008), we consider a more realistic scenario where the competition between individuals is controlled by more than one phenotypic traits. Previously, it was shown that in such systems, the number of traits or the dimension of phenotype space affects diversification, with higher dimensions leading to higher diversity (Doebeli & Ispolatov, 2017). As a community diversifies from low numbers of species, the rates of evolution and diversification slow down as the saturation level of diversity that the environment can sustain is reached. Higher rates of evolution and diversification can be reactivated only when the level of saturation decreases, which can happen with aromorphosis and an extension of the phenotypic space into higher dimensions, or through catastrophic events, leading to mass extinction (Ispolatov, Alekseeva, Alekseeva, & Doebeli, 2019).

However, it is not known which ecological and evolutionary processes unravel in such a system when external intervention, such as ongoing climate changes, continues indefinitely. A naive qualitative guess (which turns out to be correct) would be that if the rate of change associated with such an intervention is much smaller than some intrinsic adaptation rate of all species, the relative phenotypic distribution of the species would remain almost intact and all species would synchronously follow the environmental change with a certain lag. Yet it is hard to predict even qualitatively what happens when the rate of environmental changes increases, apart from the ultimate extinction of all species when environmental change is very fast. We therefore perform a systematic study of various ecological and evolutionary indicators of the evolving communities for a wide range of rates of environmental changes. To consider the most general case, we, similarly to Van Den Elzen et al. (2017), consider an asymmetric competition kernel, albeit in a different from Van Den Elzen et al. (2017) form. The evolutionary dynamics in multidimensional phenotype space with generally asymmetric competition is usually complicated (Doebeli & Ispolatov, 2017) and even unpredictable (Doebeli & Ispolatov, 2014). Thus, complimentary to many existing studies, we use a statistical approach, averaging results for each rate of environmental change over many simulated replicas.

## 2 METHODS

### 2.1 The model

Following Doebeli and Ispolatov (2017) and Ispolatov et al. (2019), we study a system that can be populated by a varying number of phenotypic species, each defined by its phenotype $\bar{x}=\left(x_{1},\ldots ,x_{D}\right)$

Here and in the following all vector notations should have bar above the variable rather than below.

in *D*‐dimensional phenotype space. In monomorphic communities consisting of a single species with phenotype $\bar{x}$, the population size of that species at ecological equilibrium is given by the carrying capacity function $K(\bar{x})$, which is assumed to have the following form:

(1) $$K\left(\bar{x}\right)=\exp\left[-\frac{\sum_{i=1}^{d}{\left(x_{i}-x_{\mathrm{ci}}\right)}^{4}}{4\sigma_{K}^{4}}\right],$$

where $\sigma_{K}$ determines the width of the carrying capacity. This function has its maximum value of 1 at the point that we call the center of the carrying capacity (CCC) ${\bar{x}}_{c}=\left(x_{c1},\ldots ,x_{\mathrm{cD}}\right)$. Thus, the population size of a monomorphic species is maximal if the phenotype of that species is equal to ${\bar{x}}_{c}$

Competition between two species with distinct phenotypes $\bar{x}$ and $\bar{y}$ is described by the competition kernel $\alpha (\bar{x},\bar{y})$, so that the competitive effect of $\bar{x}$ on $\bar{y}$ is given by

(2) $$\alpha \left(\bar{x},\bar{y}\right)=\exp\left[\sum_{i,j=1}^{D}b_{\mathrm{ij}}\left(x_{i}-y_{i}\right)\left(x_{j}-x_{\mathrm{cj}}\right)-\sum_{i=1}^{D}\frac{{\left(x_{i}-y_{i}\right)}^{2}}{2\sigma_{i}^{2}}\right].$$

There are two terms in the exponent of the competition kernel. Similarly to Van Den Elzen et al. (2017), we believe that in general the competition is nonsymmetric. Therefore, the first term represents the simplest nonsymmetric contribution to the competition, which may result in complex evolutionary dynamics, that is, cyclic or chaotic evolutionary trajectories. Since we expect the evolutionary dynamics to unravel around the CCC, we explicitly introduce the coordinates of CCC ${\bar{x}}_{c}$ into the term $\left(x_{j}-x_{\mathrm{cj}}\right)$. In our previous studies (Doebeli & Ispolatov, 2014, 2017; Ispolatov, Madhok, Madhok, & Doebeli, 2016), the CCC was fixed and positioned at zero, so the first term did not include its coordinates.

The second term in the exponent is the usual Gaussian competition kernel with width $\sigma_{i}$, reflecting the fact that species that are closer phenotypically compete more strongly with each other than species that are farther apart in phenotype space. In our simulations, we used $\sigma_{K}=1$ and $\sigma_{i}=1/2$ to ensure that the system is able to diversify from the initial state of one species to a community of coexisting phenotypes (Doebeli & Ispolatov, 2017; Ispolatov et al., 2016). Also as in Doebeli and Ispolatov (2017), the coefficients *b_ij_* of the nonsymmetric part of the competition kernel were chosen randomly from a Gaussian distribution with width 1 and zero mean (see the end of this section for how this was implemented to obtain the simulation results).

Assuming that a community comprises *m* species with phenotypes ${\bar{x}}_{r}=\left(x_{r1},\ldots ,x_{\mathrm{rD}}\right)$ in *D*‐dimensional phenotype space, where r=1,…,m is the species index, the ecological dynamics of the density *N_r_* of species *r* is given by the logistic equation

(3) $$\frac{dN_{r}\left(t\right)}{dt}=N_{r}\left(t\right)\left(1-\frac{\sum_{s=1}^{m}\alpha \left({\bar{x}}_{s},{\bar{x}}_{r}\right)N_{s}\left(t\right)}{K({\bar{x}}_{r})}\right).$$

As a result of the logistic dynamics with constant external conditions, the population of each species converges to its equilibrium size $N_{r}^{*}$. In the following, we call the state of the system, where all species have reached their $N_{r}^{*}$, as the ecological equilibrium.

In the framework of adaptive dynamics (see, for example, Diekmann, 2002; Geritz, Mesze, & Metz, 1998), evolution occurs when species, each assumed to be monomorphic in its phenotype and at the ecological equilibrium, constantly generate initially rare mutants with uniformly random phenotypes that are close to but distinct from the parental phenotype. The derivation of adaptive dynamics is based on the separation of much faster ecological and normally slower evolutionary timescales, which normally holds very well. Mutants compete with the resident community for resources and try to invade it with a per capita growth rate defined by Equation (3), where self‐competition is neglected because of mutant's rarity, $f({\bar{x}}_{1},\ldots ,{\bar{x}}_{m};{\bar{x}}_{r}^{\prime })$:

(4) $$f\left({\bar{x}}_{1},\ldots ,{\bar{x}}_{m};{\bar{x}}_{r}^{\prime }\right)=1-\frac{\sum_{s=1}^{m}\alpha \left({\bar{x}}_{s},{\bar{x}}_{r}^{\prime }\right)N_{s}^{*}}{K\left({\bar{x}}_{r}^{\prime }\right)}.$$

Here, ${\bar{x}}_{r}^{\prime }$ is the phenotype of a mutant occurring in species *r*. The function defined in Equation (4) is known as invasion fitness. If the invasion fitness is positive, the mutant population will grow in the environment set by the resident community. If the invasion fitness is negative, the mutant population goes extinct. The selection gradient ${\bar{S}}_{r}$ with components $S_{\mathrm{ri}}$ points in the direction of mutant with the highest growth rate. It is obtained by differentiating the invasion fitness with respect to the mutant phenotype and evaluating the derivative at the resident phenotype.

(5) $$S_{\mathrm{ri}}=\sum_{s=1}^{m}N_{s}^{*}\left(-\frac{1}{K({\bar{x}}_{r})}\frac{\delta \alpha \left({\bar{x}}_{s},{\bar{x}}_{r}^{\prime }\right)}{\delta x_{\mathrm{ri}}^{\prime }}{|}_{{\bar{x}}_{r}^{\prime }={\bar{x}}_{r}}+\frac{\alpha \left({\bar{x}}_{s},{\bar{x}}_{r}\right)}{K^{2}\left({\bar{x}}_{r}\right)}\frac{\delta K\left({\bar{x}}_{r}\right)}{\delta x_{\mathrm{ri}}}\right)$$

In general, the adaptive dynamics of phenotypes of a species is determined by the product of its selection gradient (5), which quantifies the selection pressure, and its mutational variance–covariance matrix, which describes the rate and size of mutations occurring in each species and their effects on the phenotypes. For simplicity, we assume there is no mutational covariance and that all traits in all species have the same mutation rate and mutational variance. To satisfy the last assumption, we implicitly rescale each direction in phenotype space such that all traits evolve at a universal rate equal to the population size times the selection gradient. Then, the adaptive dynamics of each phenotypic component $x_{\mathrm{ri}}$, i=1,…,D and r=1,…,m is then given by

(6) $$\frac{dx_{\mathrm{ri}}}{dt}=N_{r}^{*}S_{\mathrm{ri}}=\frac{N_{r}^{*}}{K\left({\bar{x}}_{r}\right)}\sum_{s=1}^{m}\left[\alpha \left({\bar{x}}_{s},{\bar{x}}_{r}\right)N_{s}^{*}\left(\sum_{j=1}^{D}b_{\mathrm{ij}}\left(x_{\mathrm{sj}}-x_{\mathrm{cj}}\right)-\frac{\left(x_{\mathrm{si}}-x_{\mathrm{ri}}\right)}{\sigma_{i}^{2}}-\frac{{\left(x_{\mathrm{ri}}-x_{\mathrm{ci}}\right)}^{3}}{\sigma_{K}^{4}}\right)\right],\;r=1,\ldots ,m;\;i=1,\ldots ,D.$$

Further details of the model, including the procedure allowing species to diversify, are presented in the next section.

So far, this model has been defined in the same way as the one in *(*Doebeli & Ispolatov, 2017; Ispolatov et al., 2016, 2019 *)*. Here, however, we introduce environmental change by assuming that over time, new phenotypes become optimal for the current state of the changing environment. The optimal phenotype in our model is defined by the position of the CCC in the phenotypic space. Thus, similarly to Johansson (2008), Van Den Elzen et al. (2017), and Jones, 2008, the environmental changes are be implemented as the motion of the CCC and the carrying capacity itself in the phenotypic space, ${\bar{x}}_{c}={\bar{x}}_{c}\left(t\right)={\bar{V}}_{c}t$. Here, the vector ${\bar{V}}_{c}$ determines the magnitude and direction of change of the CCC. While the maximum of the carrying capacity function moves in phenotype space at a constant rate ${\bar{V}}_{c}$, the general shape of the carrying capacity function, and, in particular, its width $\sigma_{K}$, stay the same in the moving frame of reference in phenotype space. In the simulations, an environmental change starts once an evolving community has reached a stationary state in the evolutionary dynamics with constant environment (Figure 2), as described in Doebeli and Ispolatov (2017) and Ispolatov et al. (2019). In the following, we denote this time as t*.

### 2.2 Simulation procedure

**FIGURE 1:**
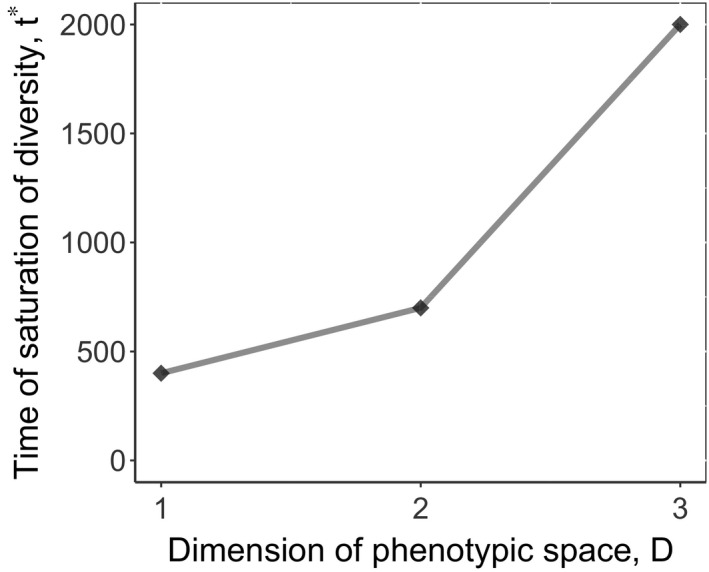
The evolutionary time t* required for diversity saturation starting from the initial condition of one randomly located species. Around the time t*, the number of species *m* equilibrates

For every dimension D=1,2,3, we prepare 30 replicate simulations, each with a distinct set of coefficients *b_ij_* and initial conditions, randomly chosen from a *D*‐dimensional Gaussian distribution with width 1 and mean 0. Every replica then evolves with nonmoving CCC positioned at zero for time t* to converge to its stationary states. The evolutionary equilibration time t* was determined empirically and was found to noticeably increase with the dimension of phenotypic space, Figure 1. Thus, in the second stage of simulation, we “reset the clock” and consider the beginning of environmental change as the new initial time *t* = 0.

**FIGURE 2:**
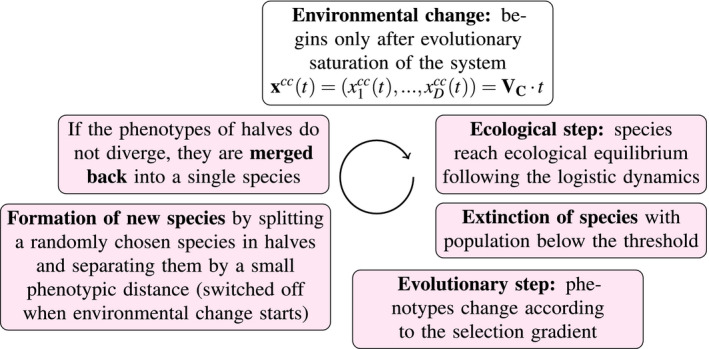
Successive steps that are iterated in the simulations. Each iterative cycle advances the evolutionary time by a small increment. The merging and splitting steps are performed once every 10 time units during the evolutionary saturation of the system. Environmental change (movement of CCC) starts only after the system converges to a steady state with saturated diversity and the formation of new species ends

A run for each replica consists of many cycles of successive steps (Figure 2), where each cycle increments the evolutionary time by a small amount. An iteration starts with the ecological dynamics, where all species reach their ecological equilibrium according to the logistic dynamics (Equation 3). Evolutionary time stays constant during this step. If a species crosses the low population limit set equal to $10^{-6}$, it is assumed to be extinct and is dropped from the system. In the next step, the phenotypes of all species evolve according to the adaptive dynamics specified in Equation (6). Phenotypic changes in a single evolutionary time step $\Delta t=10^{-2}$ are small enough to keep populations close to their ecological equilibrium (calculated in the previous step).

To model diversification, each 10 time units we split a randomly chosen species in halves separated by a very small distance Δx (normally $\Delta x=10^{-3}$). The direction of splitting is chosen randomly from the isotropic distribution. If conditions are favorable for evolutionary branching, the distance between the halves grows as a result of phenotypic dynamics, and the two “halves” become two separate species. Otherwise, that is, if the competitive interactions do not favor diversification and the halves do not move apart phenotypically, we merge them back right before the next round of splitting without any consequences to system behavior.

Both procedures of merging and splitting happen regularly every few iterations (in this order, so that split halves have time to diverge). In principle, if any two “not closely related” species come close in phenotypic distance at the time of merging, they would be merged as well, however, we have not observed such events in our simulations.

**FIGURE 3:**
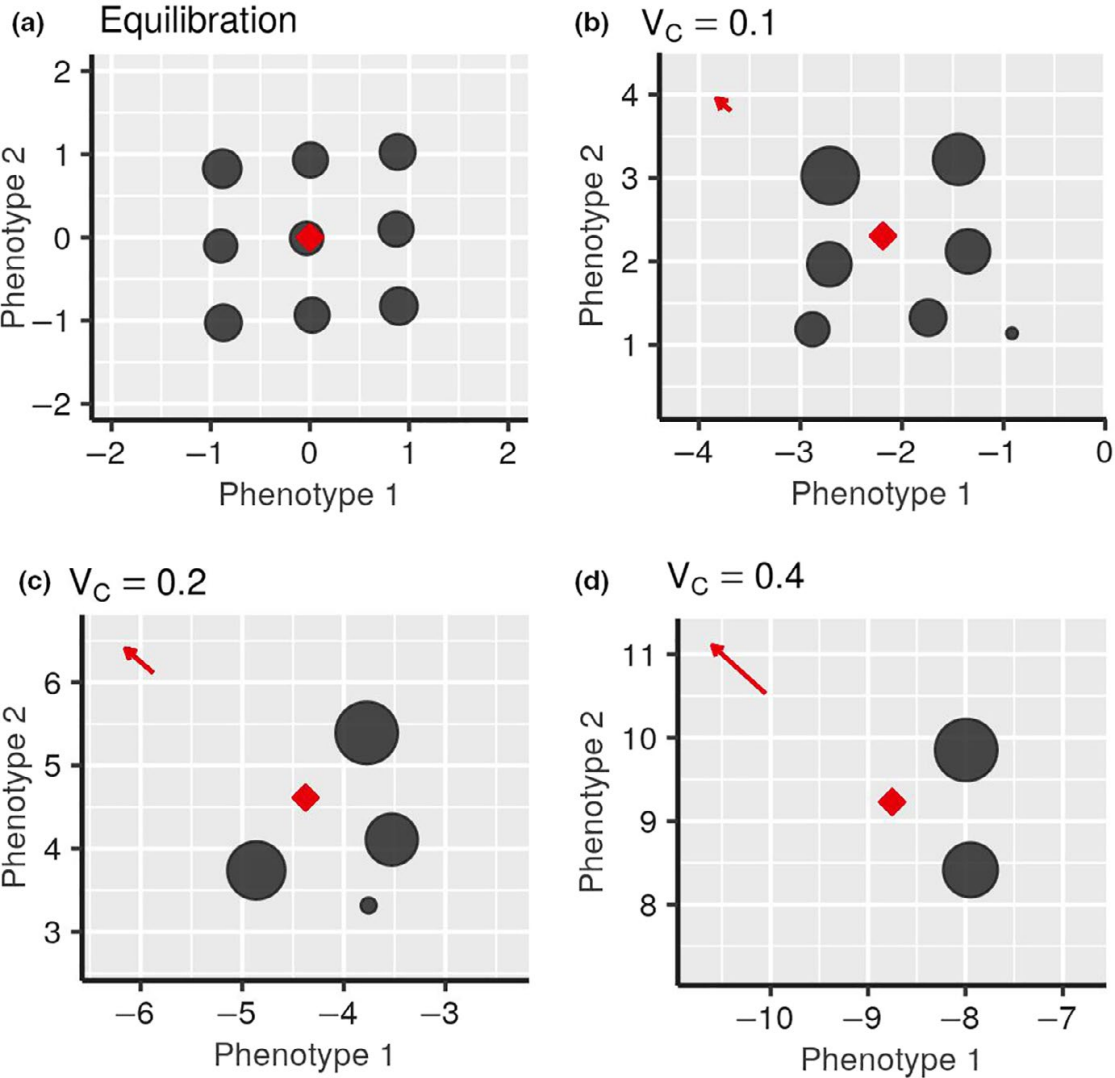
Snapshot of the saturated diversification at the beginning of environmental change *t* = 0 (a) and at *t* = 32 after the initial stage of adaptation to environmental changes of various rates (b–d) in two‐dimensional phenotypic space, $\sigma_{K}=1,\;\sigma_{i=1,2}=0.5$; The adaptation processes that led to these configurations can be seen in corresponding videos [Link], [Link], [Link], [Link] https://doi.org/10.6084/m9.figshare.12827054. In all three cases (b–d), the vector of environmental changes has the same direction to the upper left corner of the frame and is indicated by the red arrow. Dark gray circles show the location of different species in phenotype space, with the size of the circles representing the populations size, the red rhombus shows the location of the CCC. The coefficients *b_ij_* and the initial conditions can be found in Appendix S1: Section 2

In the principal part of our simulations, we analyzed how saturated systems adapt to environmental changes. For each steady state replica, a new simulation run is starting with that replica as the initial condition and under the changing environment with a given rate *V_C_* and a random direction. The rates *V_C_* are selected to cover the range between 0.05 and 2 with the step 0.05. Before the beginning of environmental changes, we merge all species, separated by phenotypic distance marginally larger than the merging distance $x=10^{-1}$ used in the diversification procedure, into distinct species, visible as circles in Figure 3 and corresponding videos. This is done to ensure that the population density *N_r_*, which controls the adaptive dynamics evolutionary speed in Equation (6), corresponds to the integral population of a species, rather than to the meta‐populations of many split “halves,” created by our diversification procedure. The biological motivation behind this final merge is that each of those individual phenotypically close “halves” can produce a mutant that could take over the whole species. Using the usually smaller individual populations of “halves” as the factor *N_r_* in Equation ((6), (7)) would have reduced the evolutionary speed and resulted in more difficult adaptation and earlier extinction. For the same reason, starting from the onset of environmental change, we switch off the procedure of formation of new species. In general, we do not expect the diversification of species under the pressure of environmental change. We further comment on this assumption in the Section 4.

**FIGURE 4:**
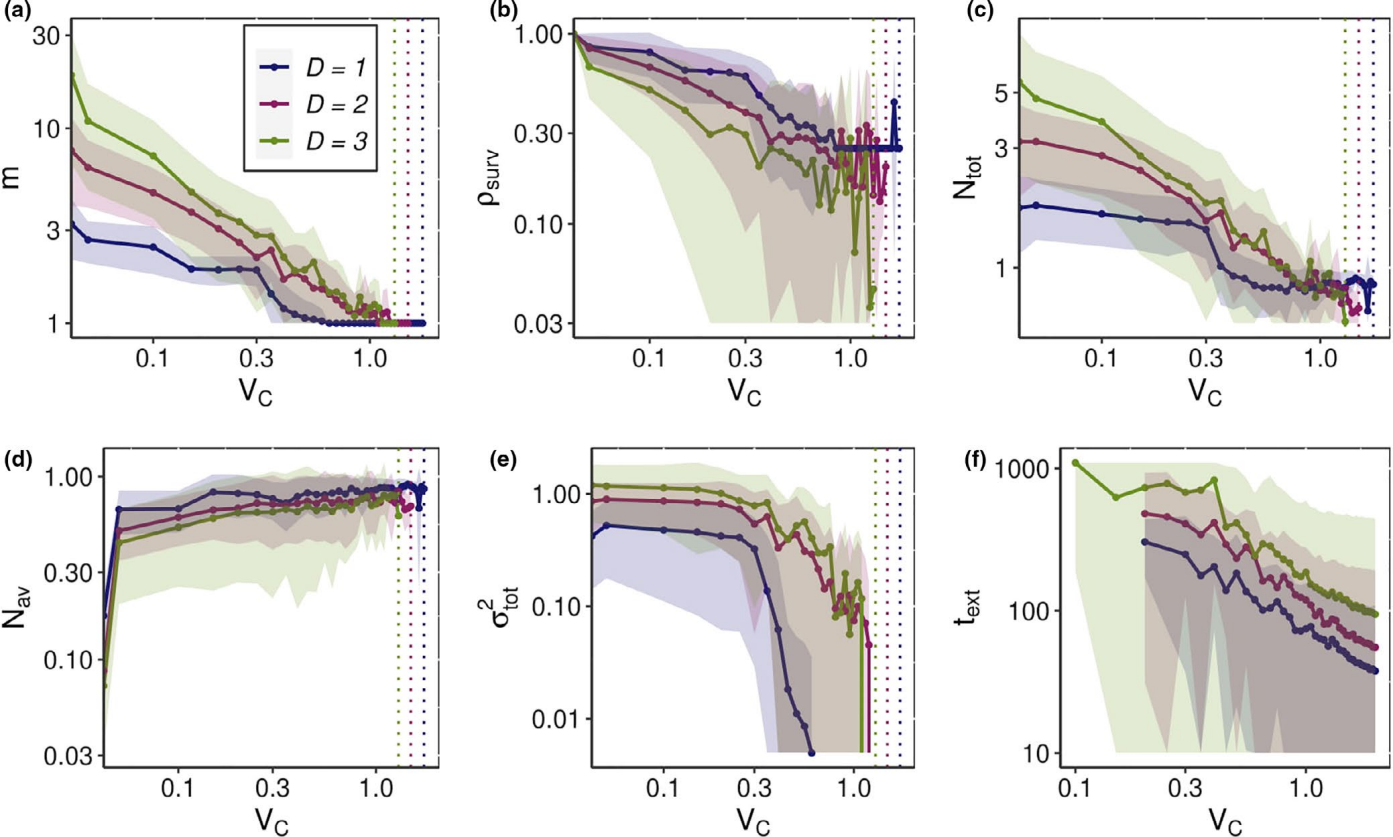
The number of species *m* (a), the fraction of species that survived till t* $\rho_{\text{surv}}$ (b), the total community population $N_{\text{tot}}$ (c), the average per species population *N*_av_ (d), the phenotypic variation across the community $\sigma_{\text{tot}}^{2}$ (e), and the time to total extinction $t_{\text{ext}}$ (f) versus the speed of environmental change *V*_C_. For each value of *V*_C_, quantities in a–e were averaged only over systems that survived under the changing environment till the final time t*. The communities that underwent extinction before t* were completely excluded from the average. The extinction time in (f) was measured only in communities that went extinct before t*. Colors indicate the dimensions of phenotype space: blue for *D *= 1, red for *D *= 2, and green for *D *= 3. Values of t* for each dimensionality of the phenotype space are presented in Figure 1. To reveal the power‐law‐like nature of many dependencies, all figures are presented in log–log scale with shadows around lines indicating standard deviations. Dotted lines represent extinction threshold $V_{C}^{\text{ext}}$ for each dimensionality, respectively

**FIGURE 5:**
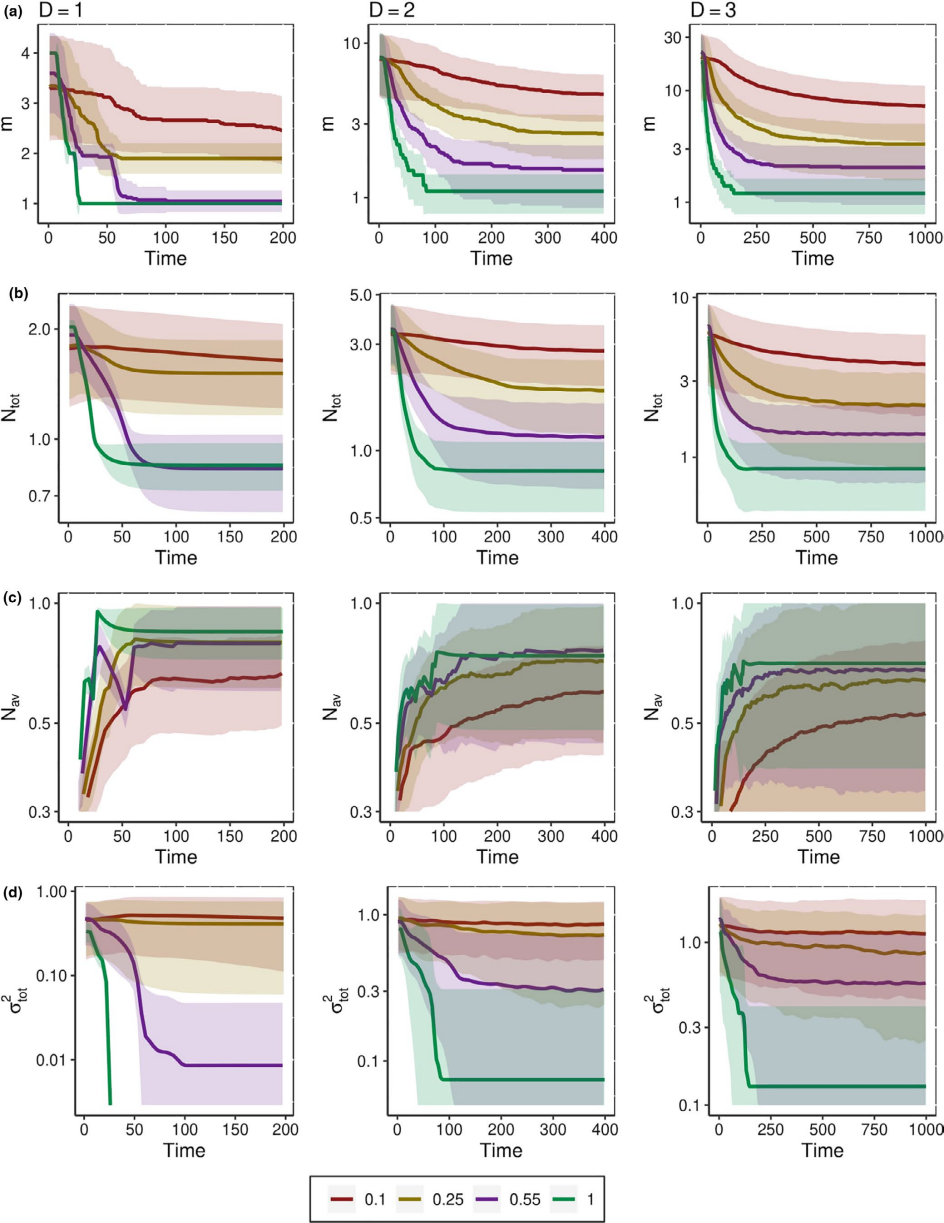
Dynamics of average number of species *m* (row a), total population $N_{\text{tot}}$ (row b), average population of a species $N_{\text{av}}$ (row c), and phenotypic variation across the community $\sigma_{\text{tot}}^{2}$ (row d) as a function of time after the onset of environmental change for 4 different values of $V_{C}$. For each value of $V_{C}$, the quantities in a–d were averaged only over systems that survived under the changing environment till the final time t*. The communities that underwent extinction before t* were completely excluded from the average. All plots are presented in semilogarithmic scale

Once started, the environmental change continues at a constant rate *V_C_* for the time t* or until all species become extinct. If a system survives the effects of CCC motion for the time t*, it usually means that it reaches a new evolutionary steady state adapted to the constant environmental change. The results for each *D* and *V_C_* are averaged over survived species communities from those 30 runs, producing statistical data shown in Figure 4 for the final state and in Figure 5 for any arbitrary time *t*.

The range of relevant CCC speed *V_C_* can be capped using the following simple analytical estimate for the maximum evolutionary speed that a single species could sustain (Johansson, 2008). The case of a single surviving species is often the final outcome of adaptation to sufficiently rapid environmental changes. If we ignore the asymmetric part of the competition kernel represented by the random coefficients *b_ij_* in Equation (6), which could either reduce or increase the strength of competition in a generally unpredictable way, the remaining adaptive dynamics becomes quite simple. The evolutionary speed $\underline{u}$ has components

(7) $$u_{i}\equiv \frac{dx_{i}}{dt}=-K\left(\bar{x}\right){\left(x_{i}-x_{\mathrm{ci}}\right)}^{3}.$$

Here, we have taken into account that the ecologically equilibrated single‐species population is equal to the corresponding carrying capacity. Assuming for simplicity that the CCC moves along the first phenotypic coordinate, we look for the maximum of *u*_1_, differentiating (7) with respect to $x_{i}-x_{\mathrm{ci}}$. The maximum is achieved at $x_{c1}-x_{1}=\left(3\right)1/4$, and the corresponding maximum speed of species motion in phenotypic space is $u_{\max}={\left(3/e\right)}^{3/4}\approx 1.08$. This sets the upper limit on the sustainable velocity of CCC, which results from a combination between two trends: A faster motion of CCC makes the species trail further behind in phenotype space, thus generating a larger selection gradient. However, the further a species trails behind the CCC, the lower is its population, which makes mutations more rare. The combination of these two trends defines the maximum velocity at which the single species can evolve, which, in other words, is the maximum velocity of CCC that a species can follow at a steady state. In reality, due to the action of the asymmetric terms in the competition kernel, and due to interspecies competition, some species go extinct below this maximum CCC velocity, while others persist even in slightly faster changing environments. The latter happens when a particular combination of randomly chosen *b_ij_* coefficients results in a stronger selection gradient in the direction of the *V_C_*.

### 2.3 Measuring properties of the system

To analyze the response of evolving communities to environmental change, in each run we measured the following system properties:

1. number of species in the system *m*;
2. total population of the system $N_{\text{tot}}=\sum_{r=1}^{m}N_{r}$;
3. average population of a species $N_{\text{av}}=\frac{1}{m}\sum_{r=1}^{m}N_{r}$;
4. phenotypic diversity of the system $\sigma_{\text{tot}}^{2}$, defined as the average square distance of the phenotypic coordinates of all species weighted by their population sizes around the center of mass of the system ${\bar{x}}^{\mathrm{mc}}=\left(x_{1}^{\mathrm{mc}},\ldots ,x_{D}^{\mathrm{mc}}\right)$, where

(8) $$x_{i}^{\mathrm{mc}}=\frac{\sum_{r=1}^{m}x_{\mathrm{ri}}N_{r}}{N_{\text{tot}}}$$

and

(9) $$\sigma_{\text{tot}}^{2}=\sum_{r=1}^{m}\frac{N_{r}}{N_{\mathrm{tot}}}\sum_{i=1}^{D}{\left(x_{\mathrm{ri}}-x_{i}^{\mathrm{mc}}\right)}^{2}.$$

The quantity $\sigma_{\text{tot}}^{2}$ reflects how widely the phenotypes of the various species are separated from the center of mass of the system. It is a measure of phenotypic diversity in a community, but should not be confused with the number of species, since the phenotype distribution in systems with smaller numbers of species can nevertheless have a higher variance if the fewer species are more spread out in phenotype space;

- fraction of survived species $\rho_{\text{surv}}=m/m_{\text{sat}}$ (10), where $m_{\text{sat}}$ is the number of species in a replica before the initiation of environmental change;
- time to extinction $t_{\text{ext}}$ defined for systems that have not persisted till t* as the time when the last species dies out.

There are two basic ways in which the above quantities can be calculated to illustrate system behavior. First, they can be evaluated at the final time t* after the onset of environmental change for many different systems with the same control parameters (e.g., the same *V_C_*). For example, we calculate the average number of coexisting species at time t*, which normally corresponds to the new evolutionary steady state adapted to the environmental change, by averaging *m* at t* for many different systems. Second, these quantities can be studied as a function of time in any given simulation run, usually starting from the initiation of the movement of CCC, that is, from the beginning of the environmental change. For example, before starting the movement of CCC, $m=m_{\text{sat}}$, that is, the number of species at saturation. Once CCC starts to move, *m* usually change, reflecting the effect of environmental change on the previously saturated community. The distribution of properties of prepared saturated systems is summarized in Figure S1.

## 3 RESULTS

We analyzed the adaptation of 30 saturated systems, properties of which were analyzed previously (Doebeli & Ispolatov, 2017; Ispolatov et al., 2019), to environmental changes of various rates. For all those systems, the environmental change in the form of a moving CCC either forces the system to adapt and converge to a new quasi‐stationary state, or it leads to extinction of the whole community.

### 3.1 Adaptation to the CCC motion and convergence to a new quasi‐stationary state

When the rate of environmental change is not too high, after a transitory adaptation the system usually converges to a new quasi‐stationary state that follows the changing environment. Properties of such a quasi‐stationary state vary depending on the rate of environmental changes. In Figure 3 there is a two‐dimensional visualization of newly formed stationary configurations of the same community, adapting to different rates of *V_C_*. For this particular example in Figure 3, we used one direction of environmental change for all presented rates of *V_C_* to make visual comparison easier, in other simulations the direction was chosen randomly each time.

The final number of species *m*, the fraction of surviving species $\rho_{\text{surv}}$ and the total population size $N_{\text{tot}}$ (Figure 4a,c) generally decrease with increasing speed of environmental changes, *V_C_*. For a given value of *V_C_*, the number of species *m* and total population $N_{\text{tot}}$ begin to decrease shortly after the onset of environmental change and after some time stabilize (despite the ongoing CCC movement), as illustrated in Figure 5a,b. The higher the *V_C_* is, the faster and larger this decrease occurs. Conversely, the average per species population $N_{\text{av}}$ in quasi‐stationary stage increases with increasing *V_C_*: as more species go extinct during the process of adaptation, the surviving ones become more ecologically successful due to experiencing less competition (Figures 4d and 5c). For large *V_C_*, we also observed scenarios, when during the adaptation $N_{\text{av}}$ first rapidly increased because of release from the competition and then fell down to a plateau due to increased distance between phenotypes of surviving species and CCC. Such a pattern can be seen on curves of $N_{\text{av}}$, which correspond to $V_{C}=1$ in D=1, and D=2 (Figure 5c).

The average value of phenotypic diversity, $\sigma_{\text{tot}}^{2}$ decreases for larger *V_C_* as well (Figure 4e), since larger rates of environmental change cause the loss of more species. Over the course of an individual simulation run, after the onset of environmental change $\sigma_{\text{tot}}^{2}$ first decreases with time and then approaches a stationary state. For very small values of *V_C_*, phenotypic dispersion may stay at the level of saturated system without environmental change or even exceed it despite a reduction in the number of species *m* (Figure 5d). Such behavior of $\sigma_{\text{tot}}^{2}$ indicates that phenotypic spread of the surviving species around the center of mass becomes wider.

Typically, the time required to reach a new evolutionarily stationary state is small relative to the saturation time t*. The higher the rate of environmental change *V_C_* is, the less time is required. The timescale of Figure 5 is two times shorter than the timescale required for initial equilibration (Figure 1).

Even for small values of *V_C_*, the entire evolving community can go extinct. Naturally, such events become more likely for larger rates of environmental changes *V_C_*.

In our simulations in any dimensionality of the phenotypic space, there is a value of *V_C_*, denoted by dotted lines on Figure 4a,e, for which none of our replicas survived. We call this value the extinction threshold, $V_{C}^{\text{ext}}$. The fraction of surviving communities as a function of *V_C_* and the extinction threshold value of *V_C_* are shown in Figure S2b of the Appendix S1. Generally, the higher the rate of environmental change, the more likely extinction occurs. Obviously, the value $V_{C}^{\text{ext}}$ may vary depending on the number of replicates and chosen t*; however, we still can compare values $V_{C}^{\text{ext}}$ between phenotype spaces of different dimensionalities, since they were obtained using the same conditions.

Times to extinction *t*_ext_ generally become shorter for larger values of *V_C_* (Figure 4f). It varies largely among replicates and is affected by particular properties of the system, such as *b_ij_* coefficients or the direction of *V_C_* vector relative to the system's phenotypic configuration.

### 3.2 The influence of phenotypic complexity on adaptation and extinction

Having more phenotypic dimensions complicates the adaptation to environmental changes. The surviving species in low‐dimensional phenotype space have larger populations *N*_av_ (Figure 4d), and even the overall population of the community *N*_tot_ of a low‐dimensional system is larger than that of a higher‐dimensional one for high rates of *V_C_* (Figure 4c). Furthermore, the time required for the community to reach the quasi‐stationary state becomes longer for higher phenotypic complexity: the parameters of systems that survive the environmental change usually reach the new steady state plateau faster in low‐dimensional systems than in high‐dimensional ones (Figure 5). However, on average, the communities with more “simple” phenotypes go extinct faster when subjected to changing environmental conditions, and we observe this effect for small and for large values of *V_C_* (Figure 4f).

Moreover, the extinction threshold becomes lower for increasing phenotypic complexity, which means that low‐dimensional communities are able to withstand rates of environmental changes that would lead to extinction of the whole community in high‐dimensional phenotype spaces (Appendix S1: Figure S2b). A mechanistic explanation for the observed reduction of the extinction threshold is that in higher dimensions, the population size *N_r_* of each species in the community is generally smaller. In higher‐dimensional systems, each species has on average more competitors due to larger number of “nearest neighbors” with slightly different phenotypes. Smaller population sizes mean lower per species mutation rates, and hence slower adaptation to changing environments (Equation 6).

To check the robustness of these results, we have repeated these simulations for fewer than 30 replicas for two other values of the width of the competition kernel, $\sigma_{i}=0.25$ and 0.75. Even though the saturated level of diversity varies strongly with $\sigma_{i}$ (Doebeli & Ispolatov, 2017), the trends shown in Figures 4 and 5 remain qualitatively unchanged.

### 3.3 Comparison to individual‐based simulations

**FIGURE 6:**
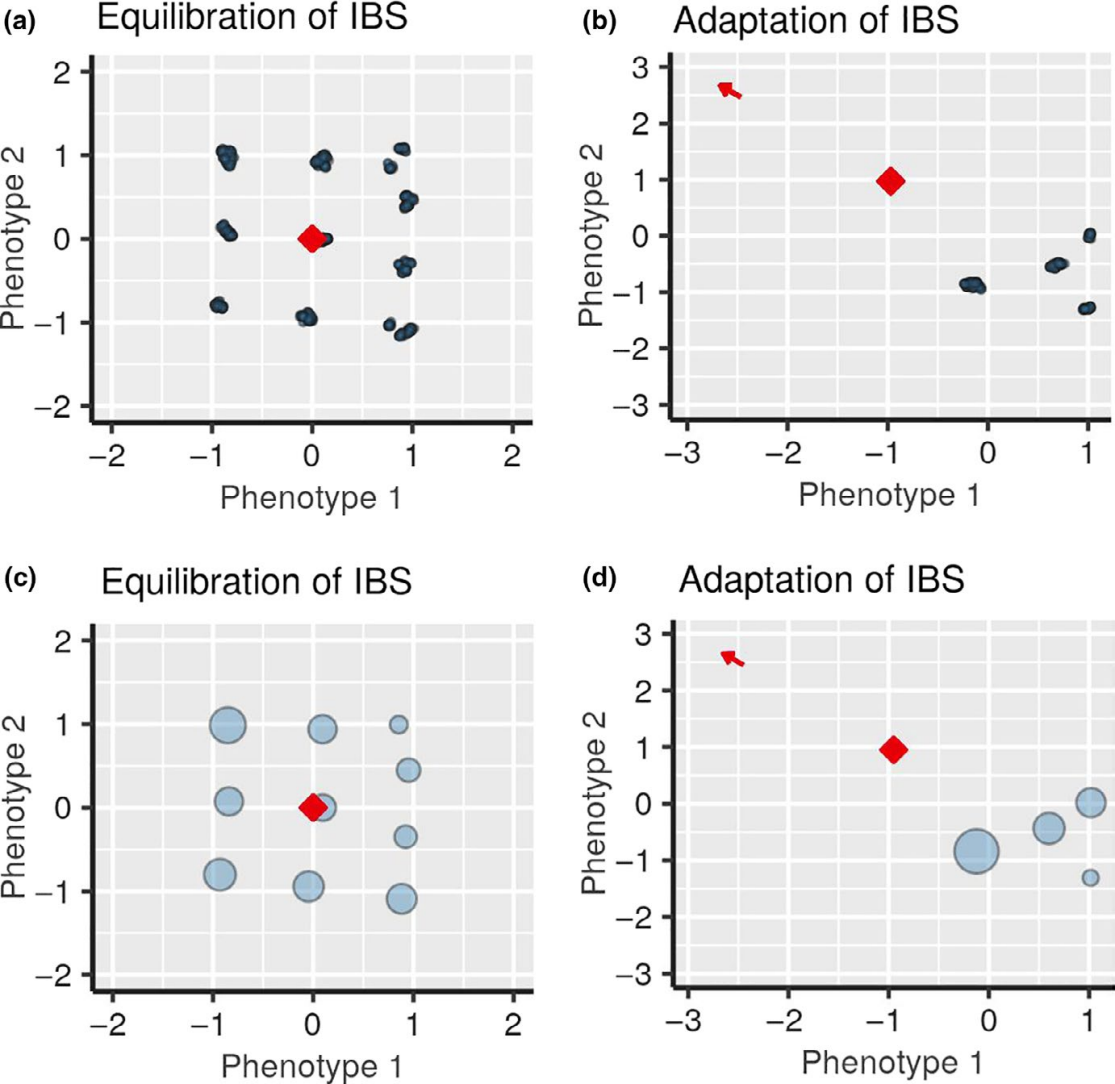
Snapshot of the saturated diversification (a, c) and adaptation to environmental changes (b, d) in individual‐based simulation. The processes that led to these configurations can be seen in corresponding videos [Link], [Link], [Link], [Link] https://doi.org/10.6084/m9.figshare.12827054. The vector of environmental changes has the direction to the upper left corner of the frame and is indicated by the red arrow. In a and b, blue circles show individuals in phenotype space, in c and d, blue circles show clustered populations of phenotypically close individuals, and with the size of the circles representing the number of individuals, the red rhombus shows the location of the CCC. The details of simulations are presented in the text. The coefficients *b_ij_* and the initial conditions can be found in Appendix S1: Section 2

To verify the robustness of our Adaptive Dynamics procedure, we, following advices from Reviewers, performed an Individual‐based simulation of the same evolving system subject to environmental change. Because individual‐based simulations are intrinsically slower than the adaptive dynamics model and repeating the statistical analysis is well beyond our computational capacity, we limited our scope to the single example shown in Figure 3. Specifically, we considered an ensemble of individuals with birth rate equal to one and death rate given by the logistic competition term in Equation (3). The carrying capacity was multiplied by the factor $K_{0}=10^{3}$, which set the scale for the total number of individuals in the community. Birth and death events were executed via the Gillespie algorithm, each offspring was offset by a randomly distributed mutation sampled from a uniform distribution with the standard deviation $\sigma_{\mu }=0.003$. The magnitude of $V_{C}$ was rescaled to take into account the actual coefficient $\sigma_{\mu }^{2}K_{0}/2$ that should have been present in Equation (6) if it were derived from the corresponding individual‐based process. Thus, to make individual‐based simulations similar to the adaptive dynamics model with $V_{C}=0.2$, the individual‐based CCC speed becomes $V_{C}^{\text{IB}}=0.0009$. In Figure 6, we show the snapshots of distributions of individuals separately (panels a and b) and clustered into species (panels c and d) immediately after equilibration (panels a and c) and after a transitory period (roughly corresponding to that in Figure 3) after the onset of environmental changes (panels b and d). A comparison between Figure 3 (panels a and c) and 6 supports the conclusion that our adaptive dynamics model is satisfactory reproducing the predictions of the “first principle” individual‐based simulations. We further comment on the correspondence between these two methods and the intrinsic limitations of our adaptive dynamics scheme in the Section 4.

## 4 DISCUSSION

We have investigated the evolutionary dynamics in logistic competition models under the continual environmental change, which was implemented by assuming that the optimal phenotype, defined by the maximum of the carrying capacity function moves at a constant speed in phenotype space. We have analyzed the effect of such environmental change on various statistical ecological and evolutionary properties of the adapting communities, depending on the rate of environmental change and on the dimension of phenotype space.

Our model is based on several assumptions, which limit us in our understanding of the full picture of adaptive evolution. For example, we assume that there is no mutational covariance and that all traits in all species have the same mutation rate. We do not distinguish phenotypic plasticity and genetic evolution, which are both important for evolution in changing environment (Ho & Zhang, 2018). In our simulations, interaction of species is limited by the competition and we do not consider numerous factors, which play an important role in evolutionary processes, such as population structure and genetic drift (Orr, 2005). We also make assumptions, which seem intrinsic to our adaptive dynamics simulation scheme, that populations of species are in their ecological equilibrium and do not diversify under the pressure of environmental changes. However, in our individual‐based simulations we observed a few times that such diversification under environmental change does happen, at least transitory. An example is shown in the videos associated with Figure 6.

These assumptions are partially justified by the fact that our principal goal is to consider the behavior of species communities on large macroevolutionary timescales. On such timescales, species undergo major phenotypic changes, which exceed the scale of phenotypic plasticity and require much more time than equilibration of species populations. A few transitory diversification events affect neither the long‐term evolution dynamics, nor the final steady state distribution of surviving species. Another justification is that we aimed to investigate global effects of environmental changes on species communities. For this goal, the implicitly performed by us rescaling of phenotypic space in a way that all traits evolve at a rate equal to the population size times the selection gradient appears to be safe and not to introduce any noticeable artifacts.

Another limitation, also intrinsic to the standard adaptive dynamics protocols, is the assumption of continuous evolution of phenotypes instead of discrete jumps caused by individual mutations. Naturally, since real mutations occur stochastically in time and carry phenotypic effects of various sizes, the adaptive dynamics methods are not well‐suited to model evolution on fine temporal and phenotypic scales. Yet the temporal and phenotypical stochasticity of mutations may also affect even the large‐scale evolutionary responses. In (Matuszewski, Hermisson, & Kopp, 2014) it was shown that an interplay between the decrease in the fraction of mutations that go in the direction “right” for adaptation with the increase of dimensionality *D*, and the increase in the “usefulness” of mutations with larger phenotypic effect (which make bigger steps to catch the CCC which moved farther because of the rarity of useful mutations) results in lesser adaptability of more complex phenotypes to environmental changes. This effect is complementary to our model and observations, yet works in the same directions.

In our models, the crucial factor determining whether an evolving community will survive in the long run is the rate of environmental change. We found that in each dimensionality of phenotype space there is a threshold rate of environmental change above which none of our replicate communities survived. However, when the rate of change is below the threshold value, many communities are able to adapt to environmental changes, and the evolving community can find a new quasi‐stationary state. Such adaptation requires much less time compared with the time it takes a community to reach the prechange level of diversity from a single ancestral species. To adapt, the phenotype of a species generally has to evolve in step with the movement of the center of the carrying capacity. Species that fail to track the carrying capacity and trail too far behind, where their carrying capacity falls significantly, suffer big population reduction and produce too few mutants to keep up with the changing environment and eventually go extinct. Similarly, species that trail too far behind their initial intraspecies phenotypic position, may experience too strong competition, which also results in the similar reduction of population, inability to produce enough mutants, and subsequent extinction. These effects were studied in great detail in simpler one‐dimensional models with 1–3 species (Johansson, 2008; Van Den Elzen et al., 2017). Our studies thus confirm that such evolutionary patterns play the essential role in the response of higher‐dimensional and higher‐diversity systems to environmental change as well. The effect of reduction in the number of species, pronounced in our simulation, was also observed in Johansson (2008) and Van Den Elzen et al. (2017), albeit on a lesser number (2–3) of initial species. These and other similarities between our results and those of Johansson (2008) and Van Den Elzen et al. (2017) signify broad universality of the observed dynamical evolutionary pattern given the differences in methodologies: We used adaptive dynamics with clonal reproduction and constant carrying capacity amplitude $K_{0}=1$, rather than individual‐based simulation with sexual reproduction and adjustable *K*_0_ used by Johansson (2008). The functional forms of the competition function and carrying capacity were different as well. Nevertheless, even though one‐dimensional systems were also included in our study, because of the difference in methodologies it is hard to go beyond a simple qualitative comparison between the presented and published results.

The rate of environmental change also affects the composition of newly formed quasi‐stationary states. The higher the rate of environmental change is, the lower is the average number of coexisting species and the total population size of surviving communities. However, surviving species get an ecological advantage: because of the reduction in the number of competitors, the populations of each surviving species can become larger than in the case of stable environmental conditions. Thus, generally the fewer species are left in the surviving community, the larger their population size becomes. This results are consistent with observations from studies of experimental evolution. In famous experiment with MEGA‐plate, the bacterial clones, which were able to adapt to higher concentration of antibiotics, became dominant in population, while their fitness, measured as the growth rate, was much smaller than the average fitness of the initial community (Baym et al., 2016).

Interestingly, phenotypic complexity makes communities less resilient to changes in the environment. In high‐dimensional phenotypic spaces, surviving systems require more time to adapt, and hence are less able to resist changes. Hence, the extinction threshold, below which no systems survive, appears at lower rates of environmental change. According to our previous studies of macroevolutionary processes (Doebeli & Ispolatov, 2014, 2017; Ispolatov et al., 2019), “complex phenotypes” have smaller populations and lower rates of evolution, which makes adaptation challenging. In these models, evolution under constant environmental conditions results in gradual expansion of phenotypic space, and more complex, higher‐dimensional phenotypes evolve only once diversity in lower dimensions has saturated (Ispolatov et al., 2019). Conversely, changing environments tend to reduce the average number of phenotypic dimensions in biological systems, since less complex species are more likely to survive.

The evolving communities that have become less diverse due to the environmental change will rediversify and reach previous saturation levels if the environment ceases to change. This happens via the same scenario as the initial diversification, illustrated, for example, in Figure 3 and corresponding videos. However, despite the similarity between the new phenotypic composition and the one existing before the onset of environmental change, the genealogical history and composition of the rediversified community may be very different from the composition of the community that existed before the environmental change was initiated.

Thus, such periods of reduction of diversity caused by environmental changes were probably followed by periods of resaturation and resulted in evolution of entirely novel phenotypes. In terms of our model of evolution of complex phenotypes (Doebeli & Ispolatov, 2017; Ispolatov et al., 2019), where species have a choice to diversify in the same phenotypic space or inhabit a new phenotypic dimension, such environmental changes would then likely generate new bouts of rapid diversification leading to saturated communities occupying higher‐dimensional phenotype spaces. We plan to include such dramatic evolutionary events in our future models of evolution of complex phenotypes (Ispolatov et al., 2019).

## CONFLICT OF INTEREST

None declared.

## AUTHOR CONTRIBUTION

**Evgeniia Alekseeva:** Conceptualization (supporting); Data curation (lead); Formal analysis (lead); Funding acquisition (equal); Investigation (lead); Methodology (equal); Project administration (equal); Resources (equal); Software (lead); Supervision (supporting); Validation (equal); Visualization (lead); Writing‐original draft (lead); Writing‐review & editing (lead). **Michael Doebeli:** Conceptualization (equal); Data curation (supporting); Formal analysis (equal); Funding acquisition (equal); Investigation (equal); Methodology (equal); Project administration (equal); Resources (equal); Software (supporting); Supervision (supporting); Validation (equal); Visualization (supporting); Writing‐original draft (equal); Writing‐review & editing (equal). **Iaroslav Ispolatov:** Conceptualization (equal); Data curation (supporting); Formal analysis (equal); Funding acquisition (equal); Investigation (equal); Methodology (equal); Project administration (equal); Resources (equal); Software (equal); Supervision (equal); Validation (equal); Visualization (supporting); Writing‐original draft (equal); Writing‐review & editing (equal).

## Supporting information

Figure S1

Figure S2

Figure S3

Figure S4

Video S1

Video S2

Video S3

Video S4

Appendix S1

## Data Availability

Codes and averaged results of simulations are available at https://github.com/EvgeniiaAlekseeva/Climate.
