# Supplementary figures and images for: Evolutionary adaptation of high‐diversity communities to changing environments
Source: Ecol Evol. 2020 Oct 13;10(21):11941–53. doi: 10.1002/ece3.6695 (PMC7663975; doi:10.1002/ece3.6695)

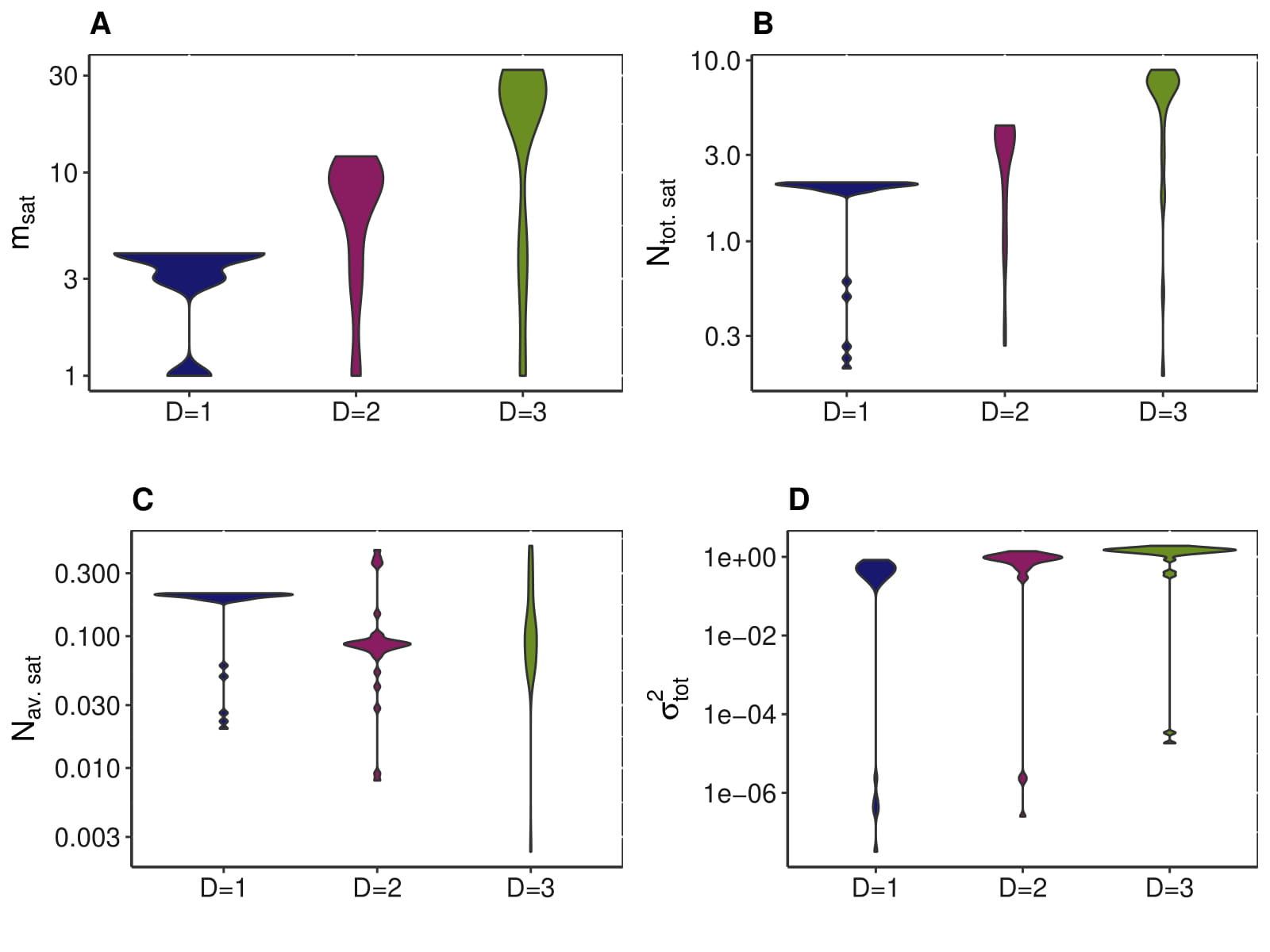

Supplement: Supplementary file 1 — Figure S1 [file ECE3-10-11941-s001.jpeg]

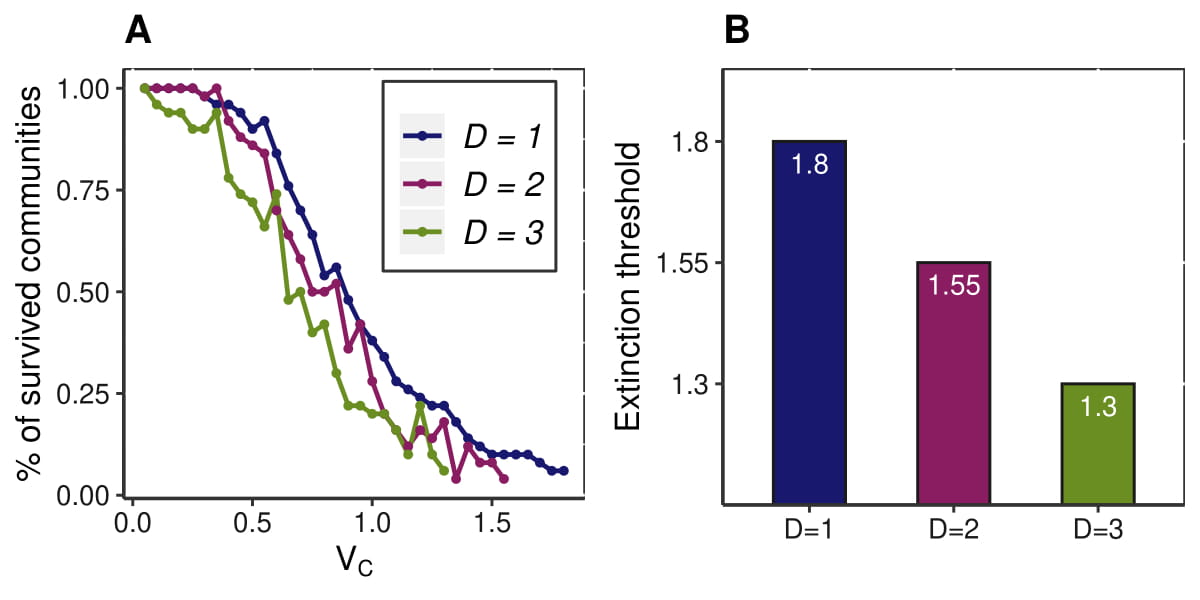

Supplement: Supplementary file 2 — Figure S2 [file ECE3-10-11941-s002.jpeg]

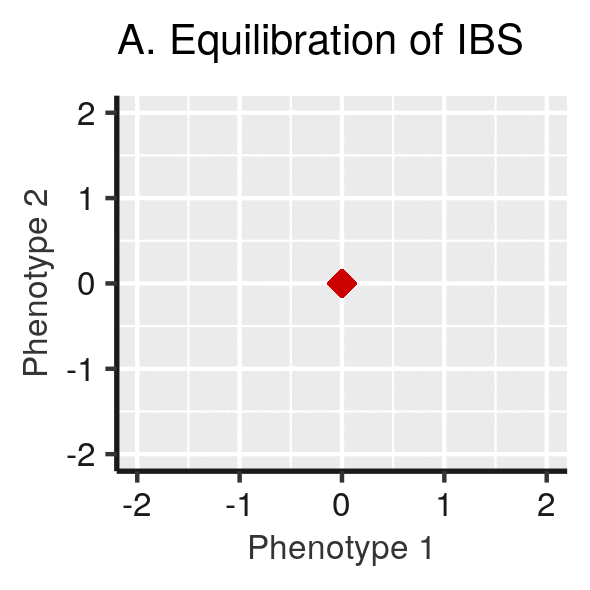

Supplement: Supplementary file 3 — Figure S3 [file ECE3-10-11941-s003.gif]

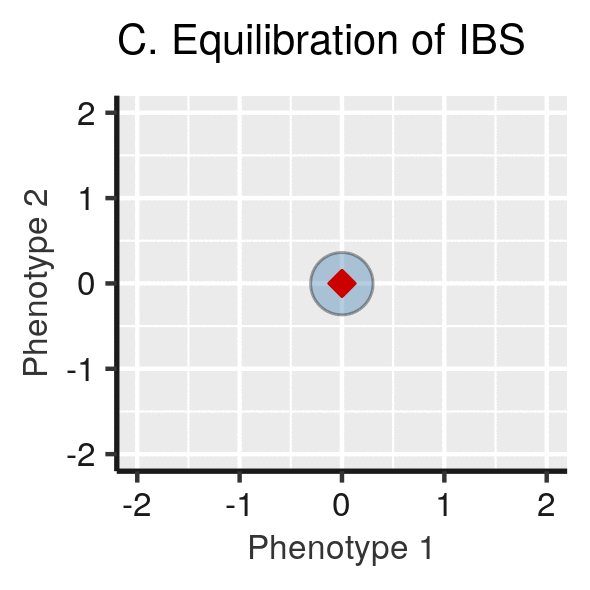

Supplement: Supplementary file 4 — Figure S4 [file ECE3-10-11941-s004.gif]

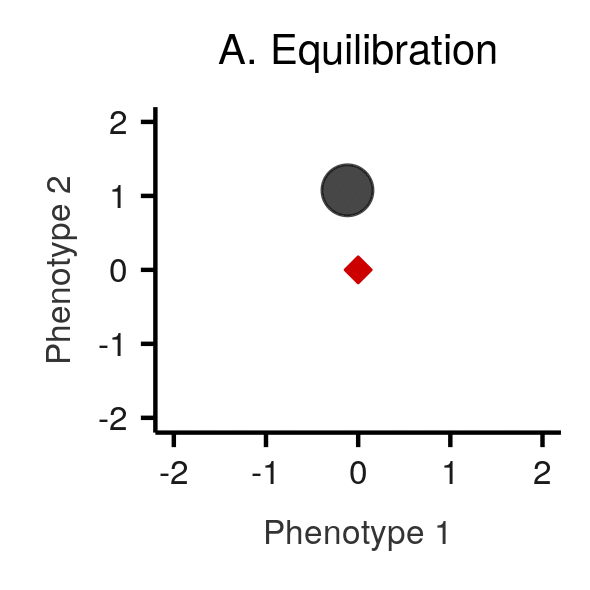

Supplement: Supplementary file 5 — Video S1 [file ECE3-10-11941-s005.gif]

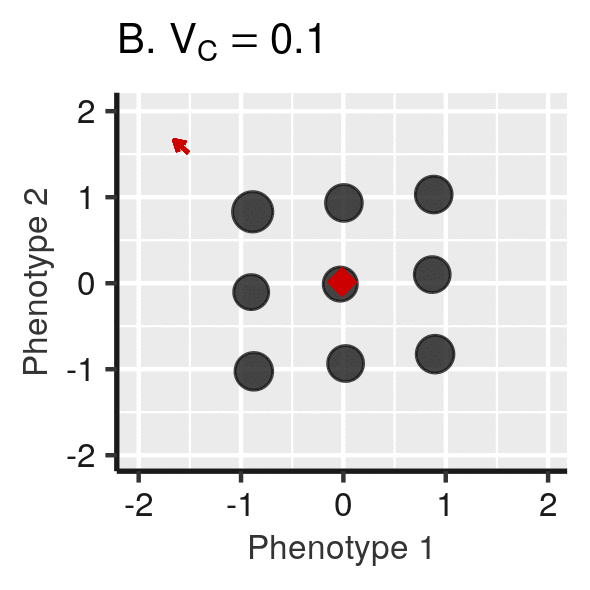

Supplement: Supplementary file 6 — Video S2 [file ECE3-10-11941-s006.gif]

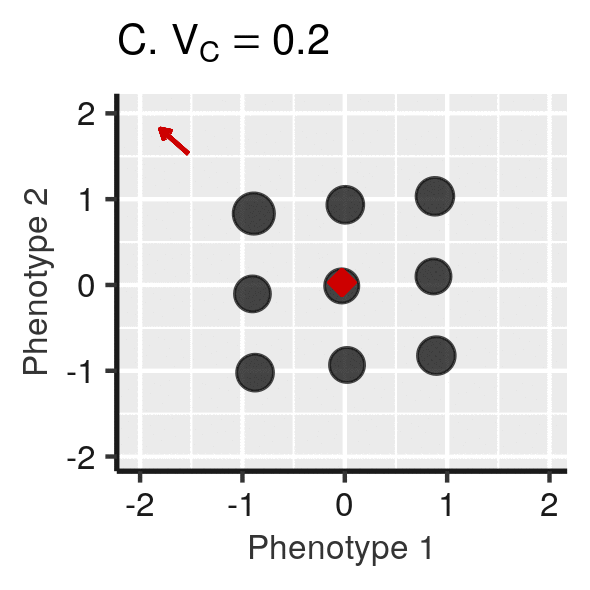

Supplement: Supplementary file 7 — Video S3 [file ECE3-10-11941-s007.gif]

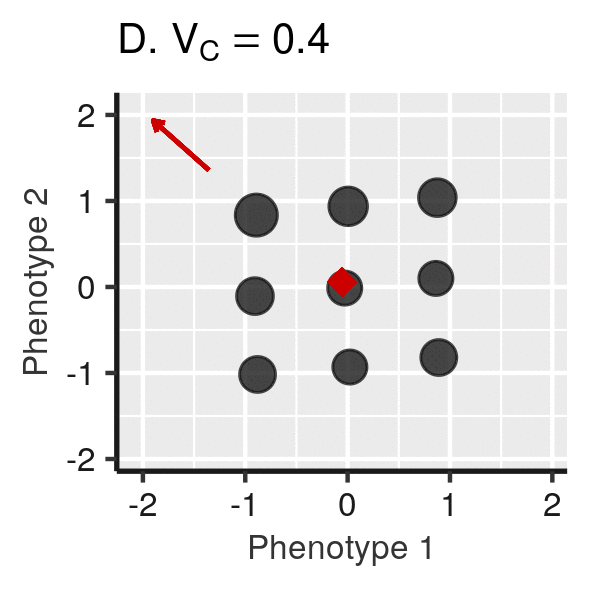

Supplement: Supplementary file 8 — Video S4 [file ECE3-10-11941-s008.gif]
