# Supplementary material for: Evolutionary adaptation of high‐diversity communities to changing environments
Source: Ecol Evol. 2020 Oct 13;10(21):11941–53. doi: 10.1002/ece3.6695 (PMC7663975; doi:10.1002/ece3.6695)
Supplement: Supplementary file 9 — Appendix S1 [file ECE3-10-11941-s009.docx]

**Appendix**

**1. Properties of saturated systems**

**Figure S1.** Distribution of properties of 30 saturated systems for each dimensionality of phenotypic space, prepared for the simulation run with the effect of environmental changes: number of species $m_{sat}$ (**A**), total community population $N_{tot.sat}$ (**B**), average per species population $N_{av.sat}$ (**C**) and the phenotypic variation across the community $\sigma_{tot}^{2}$(**D**).

**2. Visualized simulation run**

Visualized simulations, presented in Fig.1 and available for download [here](https://drive.google.com/drive/u/0/folders/1-DJQyivJb2bkO6x_ZhrzHVaF1EkMXord) have the following parameters and initial conditions:

(**A**): Diversity saturation

Simulations starts from a single species with population $N=[0.3]$ and phenotypic coordinates

$$x = [-0.1070 1.4071]$$

$$b = [0.1435 -0.0197; -0.4289 0.3450]$$

(**B**, **C**, **D**): Adaptation to environmental changes

Simulations starts from the steady state of a simulation (**A**), consisting of $m=9$ species with populations

$N=[0.4819 0.4923 0.4501 0.5187 0.5092 0.4704 0.47321 0.4432 0.4726]$ and phenotypic coordinates

$$\underline{x}=[0.8842 1.0289; -0.8727 -1.0268; -0.8995 -0.1023; -0.8844 0.8286;$$

$$0.8970 -0.8258; 0.0216 -0.9344; 0.0050 0.9326; -0.0319 -0.0096;$$

$$0.8693 0.1011]$$

**3. Threshold value of** $\boldsymbol{V}_{\boldsymbol{C}}$

**Figure S2**. **A**: The fraction of systems with at least one surviving species from the sample of 30 saturated systems vs. the speed of CCC motion for three dimensions of phenotypic space; **B**: Extinction threshold $V_{C}^{ext}$for three dimensionalities of phenotypic space.
